# Raman–deuterium isotope probing to study metabolic activities of single bacterial cells in human intestinal microbiota

**DOI:** 10.1111/1751-7915.13519

**Published:** 2019-12-10

**Authors:** Yi Wang, Jiabao Xu, Lingchao Kong, Tang Liu, Lingbo Yi, Hongjuan Wang, Wei E. Huang, Chunmiao Zheng

**Affiliations:** ^1^ School of Environment Harbin Institute of Technology Harbin 150090 China; ^2^ Guangdong Provincial Key Laboratory of Soil and Groundwater Pollution Control School of Environmental Science and Engineering Southern University of Science and Technology Shenzhen 518055 China; ^3^ Department of Engineering Science University of Oxford Parks Road Oxford OX1 3PJ UK; ^4^ Health Time Gene Institute Shenzhen 518000 China

## Abstract

Human intestinal microbiota is important to host health and is associated with various diseases. It is a challenge to identify the functions and metabolic activity of microorganisms at the single‐cell level in gut microbial community. In this study, we applied Raman microspectroscopy and deuterium isotope probing (Raman–DIP) to quantitatively measure the metabolic activities of intestinal bacteria from two individuals and analysed lipids and phenylalanine metabolic pathways of functional microorganisms *in situ*. After anaerobically incubating the human faeces with heavy water (D_2_O), D_2_O with specific substrates (glucose, tyrosine, tryptophan and oleic acid) and deuterated glucose, the C–D band in single‐cell Raman spectra appeared in some bacteria in faeces, due to the Raman shift from the C–H band. Such Raman shift was used to indicate the general metabolic activity and the activities in response to the specific substrates. In the two individuals' intestinal microbiota, the structures of the microbial communities were different and the general metabolic activities were 76 ± 1.0% and 30 ± 2.0%. We found that glucose, but not tyrosine, tryptophan and oleic acid, significantly stimulated metabolic activity of the intestinal bacteria. We also demonstrated that the bacteria within microbiota preferably used glucose to synthesize fatty acids in faeces environment, whilst they used glucose to synthesize phenylalanine in laboratory growth environment (e.g. LB medium). Our work provides a useful approach for investigating the metabolic activity *in situ* and revealing different pathways of human intestinal microbiota at the single‐cell level.

## Graphical abstract

This study aims at developing a new approach to investigate the microbial metabolism in human intestine *in situ* and revealing different pathways of human intestinal microbiota at the single‐cell level.

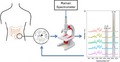

## Introduction

The human gut microbiota consists of up to 3.8 × 10^13^ microbes, which is closer to the number of human cells of 70 kg males (Sender *et al.*, 2016) and contains ~100 times more genes than the human's genome(Backhed *et al.*, 2005; Gill *et al.*, 2006; Ley *et al.*, 2006; Thursby and Juge, 2017). The composition and activity of the human intestinal microbes are associated with diseases, such as obesity, inflammatory bowel disease, fatty liver disease and hypertension, and human health states such as body temperature regulation, reproduction and tissue growth (Nicholson *et al.*, 2012). With increasing evidence to show the importance of human intestinal microbes, the advances in several methodologies have accelerated the research of profiling and characterizing the complex ecosystems in the gut. The most traditional culture‐based methods to study microbiota can be biased because only 20–40% of the human intestinal microbiota can be cultured (Amann *et al.*, 1995; Thursby and Juge, 2017) and many species are not yet isolated for cultivation (Amann *et al.*, 1995). It is likely that the isolated bacteria from their natural habitats would alter metabolism and physiology states. Culture‐independent methods have entered the centre‐stage of microbiota study, including the omics technologies and flow cytometry. Metagenomic sequencing of either 16S rRNA or shotgun sequencing provides insights in estimating the composition, diversity and functional genes of the complex gut microbial communities (Qin *et al.*, 2010; Human Microbiome Project, 2012). However, cell individuality, heterogeneity and cell‐to‐cell interaction can be lost in the bulk omics approaches. Single‐cell analysis is becoming important to detect, identify, count and characterize single bacterial cells at biological context or heterogeneity in a mixed microbial population (Shapiro, 2000). Flow cytometry is a single‐cell technique that can detect single cells based on cell properties such as membrane integrity, polarity or the relative molecular content with fluorescent probes (Joux and Lebaron, 2000; Ben‐Amor *et al.*, 2005; Maurice *et al.*, 2013; Zimmermann *et al.*, 2016). However, it is difficult to detect the intrinsic biochemical information and bacterial metabolic functions within the microbiota in response to a particular substrate or drug.

Single‐cell Raman microspectroscopy (SCRM) is a label‐free and non‐destructive vibrational spectroscopy that can obtain a molecular vibrational profile of a cell. A single‐cell Raman spectrum (SCRS) can be regarded as the biochemical fingerprint of a cell, revealing intrinsic information such as essential biomolecules, specific biomarkers and metabolic states of cells (Li *et al.*, 2012; Naemat *et al.*, 2016; Xu *et al.*, 2017; Lee *et al.*, 2019). Raman spectroscopy combined with deuterium isotope probing (Raman–DIP) is able to probe the metabolism of single cells *in situ* (Huang *et al.*, 2007; Henk‐Jan *et al.*, 2008; Berry *et al.*, 2015; Xu *et al.*, 2017). When bacteria are cultivated with heavy water (D_2_O) or a deuterated substrate, the active cells will incorporate deuterium into their biomass via the action of H/D exchange reactions. The C–D Raman band is distinctive at 2000–2300 cm^−1^ in SCRS and the intensity can be used as a universal Raman marker to determine metabolic activity of single cells (Berry *et al.*, 2015). Several studies have investigated the active microbial cells from pure culture (Xu *et al.*, 2017), river water (Xu *et al.*, 2017), soil (Cui *et al.*, 2018; Lee *et al.*, 2019) and mice faecal samples (Berry *et al.*, 2015) by using Raman–DIP. Different substrates from daily dietary intake shape the overall composition and functions of the intestinal microbial community, and some of the substrates are the key factors in the colonization of specific gut bacterial species (Dai and Walker, 1999). However, substrate utilization and activities as an individual and as a community in the complex human gut are still elusive.

In this study, we used Raman–DIP to study the metabolic activities and reveal their pathways of the intestinal microbiota in response to different nutrient sources such as sugar (glucose), amino acids (tyrosine and tryptophan) and fatty acids (oleic acid). By *in situ* incubation of faecal bacteria from two healthy donors with D_2_O or a combination of D_2_O with tyrosine, tryptophan or oleic acid, the metabolically active bacterial populations were identified in relation to their functions in the complex microbial environment. The degradation pathways of deuterated glucose by the intestinal bacteria were also investigated by calculating relative intensities of the specific Raman bands. The study of functional bacteria in the gut microbiota at the single‐cell level might lead to a deeper understanding of microbiota and help to establish foundation to personalized medicine related to gut health.

## Results and discussion

### Detecting the metabolic activities of human intestinal microbiota at the single‐cell level using Raman–DIP

**Figure 1:**
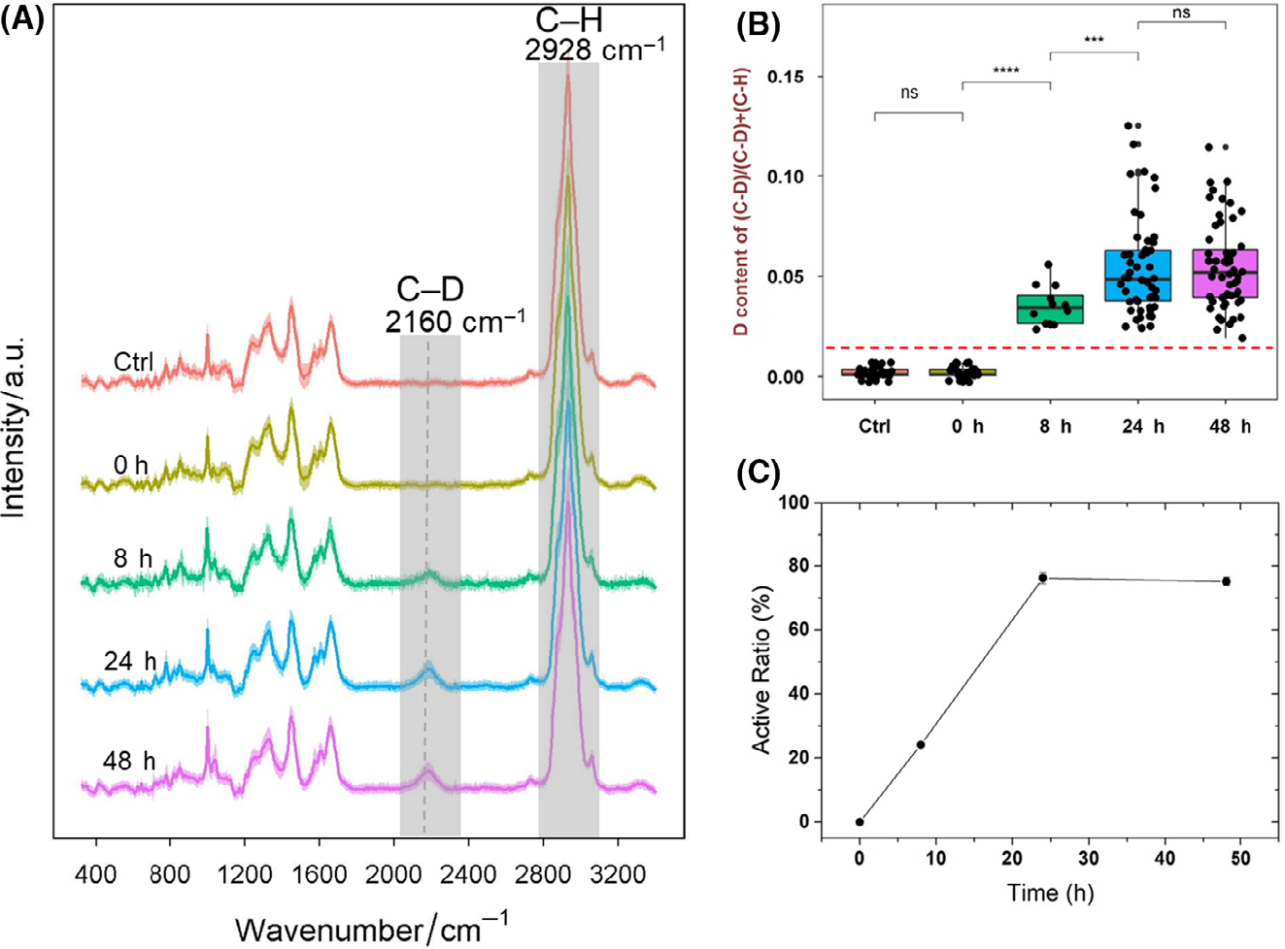
General metabolic activity of human gut bacteria at different time points from the faeces of volunteer 1. A. SCRS with the C–D band (2070–2300 cm^−1^) highlighted. Each spectrum represents an average of SCRS from 50 to 80 single cells, and the shadow represents standard deviation of SCRS. B. The intensity ratio of C–D/(C–D + C–H) in SCRS of single cells. Only SCRS of active cells were included at the time point of 8, 24 and 48 h. Student *t*‐test was used to indicate statistical differences between different time points, in which ns indicates non‐significant, *P < 0.05, **P < 0.01, ***P < 0.001, ****P < 0.0001. The threshold, defined as the mean + 3 × SD of C–D% in randomly selected cells from the control sample, is shown in red. C. The ratio of active bacteria in the total microbiota population identified by the presence of the C–D band.

Raman–DIP was applied to detect metabolic activity of single microbial cells in the human intestinal microbiota from two healthy volunteers. Figure 1A shows the averaged SCRS of 50–80 randomly selected bacterial single cells of the microbiota from volunteer one, after incubating the faeces with 40% D_2_O for 0, 8, 24 and 48 h anaerobically *in situ*. Each sample had three replicates. At 8‐h post‐cultivation, a broad Raman band appeared in the region between 2020 and 2300 cm^−1^, peaked at 2160 cm^−1^ (Fig. 1A), which is C–D stretching vibrations shifted from the C–H stretching vibrations at 2800–3200 cm^−1^ (Berry *et al.*, 2015). This shift was due to the incorporation of deuterium from D_2_O to bacterial biomass via NADPH‐mediated H/D exchange reactions in the metabolically active bacteria. The intensity of the C–D band is proportional to the general metabolic activity of bacteria (Berry *et al.*, 2015). In the control samples which the faeces incubating with H_2_O, SCRS show the absence of the C–D band (Fig. 1A).

Semi‐quantification of deuterium incorporation was calculated using the ratio of C–D/(C–H + C–D) as previously reported (Berry *et al.*, 2015) (Fig. 1B). The threshold of labelling was calculated as the mean + 3 × SD of C–D% in randomly selected cells from the control sample. A gradual increase in the C–D band was observed from 0, 8 to 24 h, indicating active metabolism of bacteria in the microbiome environment. After 24 h, the C–D band reached stationary phase, which might be due to the exhaustion of sources in the faecal environment. Hence, the time point of 24 h under anaerobic incubation at 37°C was selected in further experiments investigating the metabolism of gut microbiota.

The ratio of active bacteria in total microbiota population was decided by counting the number of bacteria showing C–D bands in SCRS from ~80 randomly selected individual cells at each time point (Fig. 1C). Around 23.5% of the population was found metabolically active at 8 h, which increased to 76.0% and 74.6% at 24 and 48 h. The temporal difference of active bacterial population indicates a variation in the metabolic rates of individual bacterial cells (Devaraj *et al.*, 2013; Maurice *et al.*, 2013), as some bacteria have faster and the others have slower rates of metabolic activities.

A previous study using cultivation method suggested that a 17% bacteria in human gut microbiota were active (Tilg and Gao, 2015). On the other hand, only 20–40% of the human intestinal microbiota have been found cultivable (Amann *et al.*, 1995). Hence, it might be difficult to use traditional cultivation‐dependent techniques to reveal the functional bacteria of the complex gut microbial community *in situ*. The population of active cells found by fluorescent probes varied from 44% to 83% depending on choices of analytical methods (Maurice *et al.*, 2013). These physiology‐based measures are often indirect to measure bacterial metabolic activity. In contrast, Raman–DIP provides an *in situ,* sensitive, semi‐quantitative and label‐free tool to distinguish an active bacterial population in the human microbiota.

### Raman–DIP detected the microbial activities in response to different nutrients

**Figure 2:**
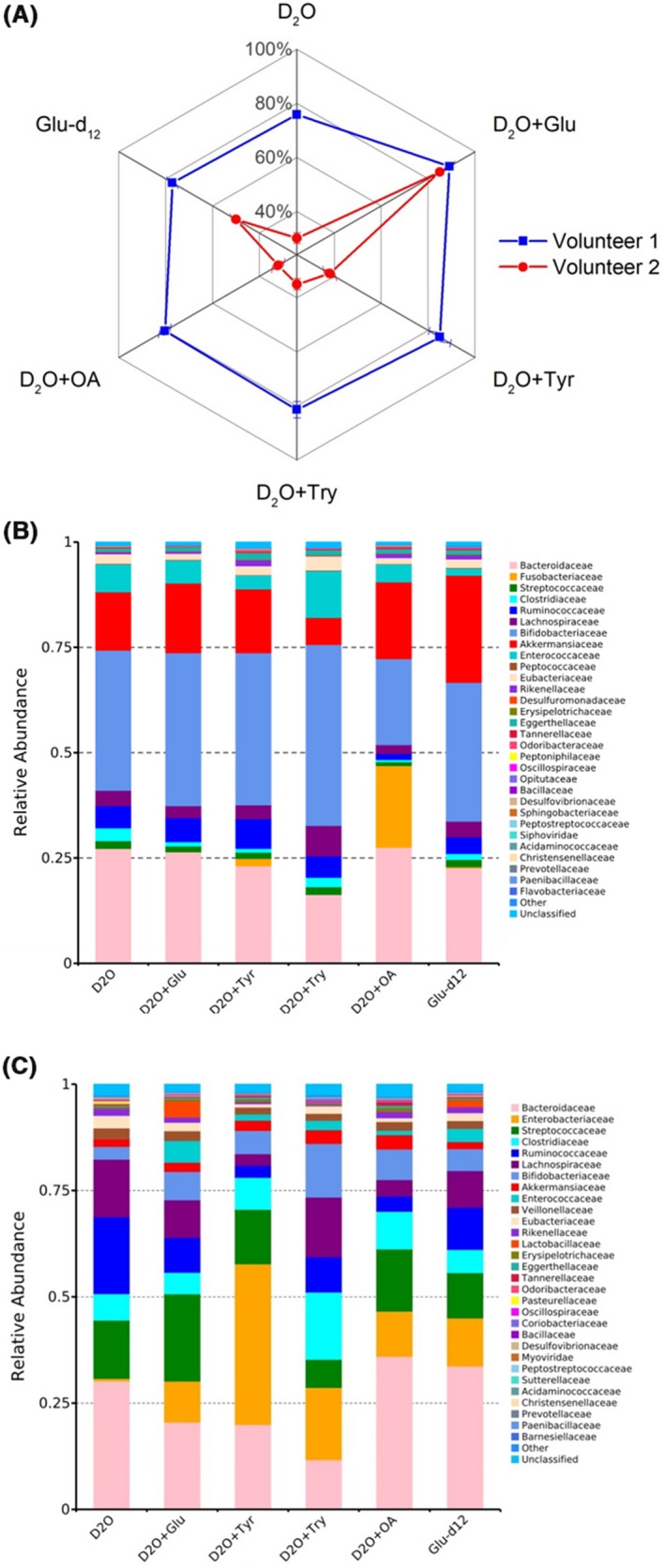
A. Percentages of the active intestinal microbiota from two healthy individuals in response to different nutrients measured by Raman–DIP. For each condition, three replicates were taken with 50–80 cells per sample. The error bar represents standard deviation of three replicates. B. and C. Structures of the microbial community (top 30 at family level) of two healthy individuals with different nutrient supplements (*E.coli* was excluded).

The degradation of various dietary components by the human microbiota in large intestine contributes nutrients and energy to the host and is important to host metabolism and immune system (Flint *et al.*, 2012). Raman–DIP was used to probe the activity of intestinal microbes in response to D_2_O, and different substrates such as glucose, tyrosine, tryptophan and oleic acid in the faeces of two volunteers (Fig. 2A). The structure of the microbiota community and the stimulated genes catabolizing the compounds was analysed in each condition by performing metagenomic sequencing (Fig. 2B,C, Figs S1 and S2). The community structures of intestinal microbes vary greatly between individuals, which is conceivable as different diet and environmental factors (Conlon and Bird, 2015). Although the two volunteers are both Chinese, volunteer 1 has been living in China and prefers Chinese food and rice, while volunteer 2 has been living in the United Kingdom for more than 7 years and prefers western food, fresh vegetables and fruit. Raman–DIP analysis at 24 h reveals that the overall active ratios in volunteer 1 and 2 were 76 ± 1.0% and 30 ± 2.0% respectively, without any substrate supplement (Fig. 2A), because there are residual nutrients remained in faeces, which can be used for microbial metabolism.

Interestingly, addition of 10 mM glucose significantly enhanced the active ratios to 90 ± 1.4% and 85 ± 1.4%, comparing to the controls of 76 ± 1.0% and 30 ± 2.0% in two volunteers after 24 h. In contrast, the supplement of tyrosine, tryptophan and oleic acid did not show such strong stimulation effect in both volunteers (Fig. 2A). Glucose might be the favourable carbon source by intestinal bacteria, thus enhancing the active ratio by the greatest extent (Schneckenberger *et al.*, 2008). This is in a good agreement with the previous study in mice showing the active ratio increase from 9% to 81% with glucose addition (Berry *et al.*, 2015).

Tyrosine and tryptophan are two essential amino acids found in many dietary proteins and protein‐based food, both of which contribute uniquely to the gut‐brain axis as the sole precursors of dopamine and serotonin (Rowland *et al.*, 2018). Tyrosine and tryptophan were found to slightly enhance the percentage of the active community to 85 ± 4.6% and 81 ± 3.1% (comparing to the non‐supplement control of 76 ± 1.0%, *P* < 0.05); and 38 ± 2.6% and 35 ± 2.1% (comparing to the non‐supplement control of 30 ± 2.0%, *P* < 0.05) to in two volunteers respectively (Fig. 2A). A previous *in vitro* culture‐based study suggested that tyrosine had a stronger effect on modifying bacterial active ratio compared with tryptophan (Smith and Macfarlane, 1998). This might be due to less energetic requirement in the breakdown of the phenol functionality in tyrosine compared with that of the indole moiety on tryptophan (Schneckenberger *et al.*, 2008; Davila *et al.*, 2013).

Oleic acid is a common monounsaturated fat (MUFA) regularly found in diet as part of animal fats and vegetable oils. MUFA consumption has been associated with several health benefits such as decreased low‐density lipoprotein cholesterol and reduction in blood pressure (Teres *et al.*, 2008). Supplements of palm and olive oil have been found to largely shape the gut microbiota (Candido *et al.*, 2018). With addition of pure oleic acid, a slightly higher percentage of the community was active (80 ± 2.5% and 32 ± 2.6%), suggesting use of MUFA as energy and nutrient sources by certain members of the community.

Randomly measured bacteria show that the active ratios of bacteria response to glucose‐d_12_ and 80 ± 2.0% and 50 ± 2.0% of intestinal bacteria were able to directly utilize glucose in the two volunteers (Fig. 2A). The higher active ratios of the metabolically active cells in the case of D_2_O + glucose (90 ± 1.4% and 85 ± 1.4% in two volunteers) suggest that some detected metabolic activity in intestinal bacteria could be caused by cross‐feeding between glucose‐user and other bacteria (Fig. 2A). This phenomenon is consistent with the commensalism among microorganisms and the priming effect in soil. Early studies have confirmed that the effect of commensals is common in human guts (Ventura *et al.*, 2012), for example obligate anaerobes can grow in the colon when oxygen is used up by the facultative anaerobic bacteria (Conway and Cohen, 2015).

### Raman–DIP revealed glucose metabolism to fatty acids in gut microbiota

Raman–DIP using glucose‐d_12_ has previously been applied to study the metabolic pathway of carbon sources in pure cultures of *Escherichia coli* and *Pseudomonas putida* (Xu *et al.*, 2017). Here, we used this technique to study the metabolic pathway of glucose by bacteria in the human microbiota.

**Figure 3:**
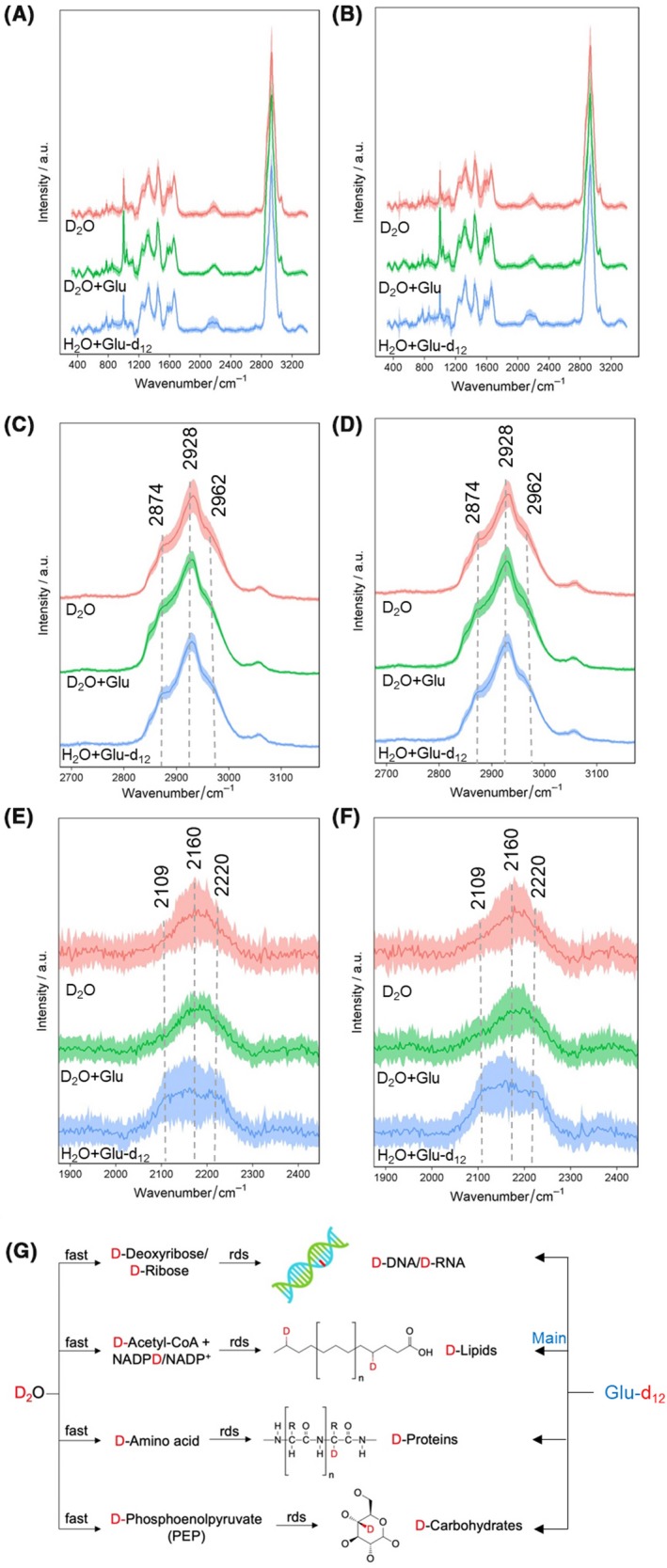
SCRS of human gut bacteria cultured *in situ* with D_2_O, D_2_O + 10 mM glucose and H_2_O + 10 mM glucose‐d_12_ of (A) volunteer 1 and (B) volunteer 2. Each spectrum represents an average of SCRS from 50 to 80 single cells, and the shadow represents standard deviation. Enlarged view of the C–H band (2800–3200 cm^−1^) consisted of DNA (2962 cm^−1^), proteins (2928 cm^−1^) and lipids (2874 cm^−1^) of (C) volunteer 1 and (D) volunteer 2. And the enlarged view of and the C–D band (2070–2300 cm^−1^) consisted of DNA (2220 cm^−1^), proteins (2160 cm^−1^) and lipids (2109 cm^−1^) of (E) volunteer 1 and (F) volunteer 2. G. Intracellular D incorporation from D_2_O or glucose‐d_12_ in gut bacteria. D incorporation from D_2_O proceeds via the integration of the labelled molecular precursors into various biomolecules, as the rate‐determining steps (rds). On the other hand, D incorporation from glucose‐d_12 _is found mainly contributed by the lipid synthesis.

Figure 3A,B shows the averaged SCRS of microbial cells from two volunteers cultured *in situ* with D_2_O, D_2_O + 10 mM glucose and H_2_O + 10 mM glucose‐d_12_ respectively. The broad C–D band was observed in all conditions, due to incorporation of intracellular D from either D_2_O or deuterated carbon source. D_2_O diffuses freely into the cells, and D atoms are bonded to carbon atoms through various enzymatic reactions (Miyagi and Kasumov, 2016). These processes label precursors of all kinds of biopolymers, that is amino acids for proteins, acetyl‐CoA/NADAH for fatty acids and deoxyriboses for DNA (Fig. 3G). Therefore, the deuterium incorporation via D_2_O can be compared with the incorporation from specific substrates to investigate unique pathways.

Figure 3C–F shows the enlarged views at the C–H and C–D regions in Fig. 3A,B, both of which have been demonstrated to consist of distinct biocomponents within a cell. In Fig. 3C,D, the C–H band can be decomposed to C–H in three cellular components of DNA (2962 cm^−1^), proteins (2928 cm^−1^) and lipids (2874 cm^−1^) (Chen *et al.*, 2015; Shi *et al.*, 2018). Accordingly, the C–D shift in Fig. 3C,D was associated with the C–D bond‐containing DNA (2220 cm^−1^), proteins (2160 cm^−1^) and lipids (2109 cm^−1^) (Chen *et al.*, 2015; Shi *et al.*, 2018). A distinctive pattern of the C–D band was observed using glucose‐d_12_ compared with D_2_O, with a visibly higher contribution by the lipid component at 2109 cm^−1^ (Fig. 3E,F). It should be noted that nearly all active cells exhibit a similar pattern of the higher intensity at 2109 cm^−1^, suggested by a previous study as the vibrational position of a fully deuterated palmitic acid molecule (Zhang *et al.*, 2011; Stiebing *et al.*, 2014).

**Figure 4:**
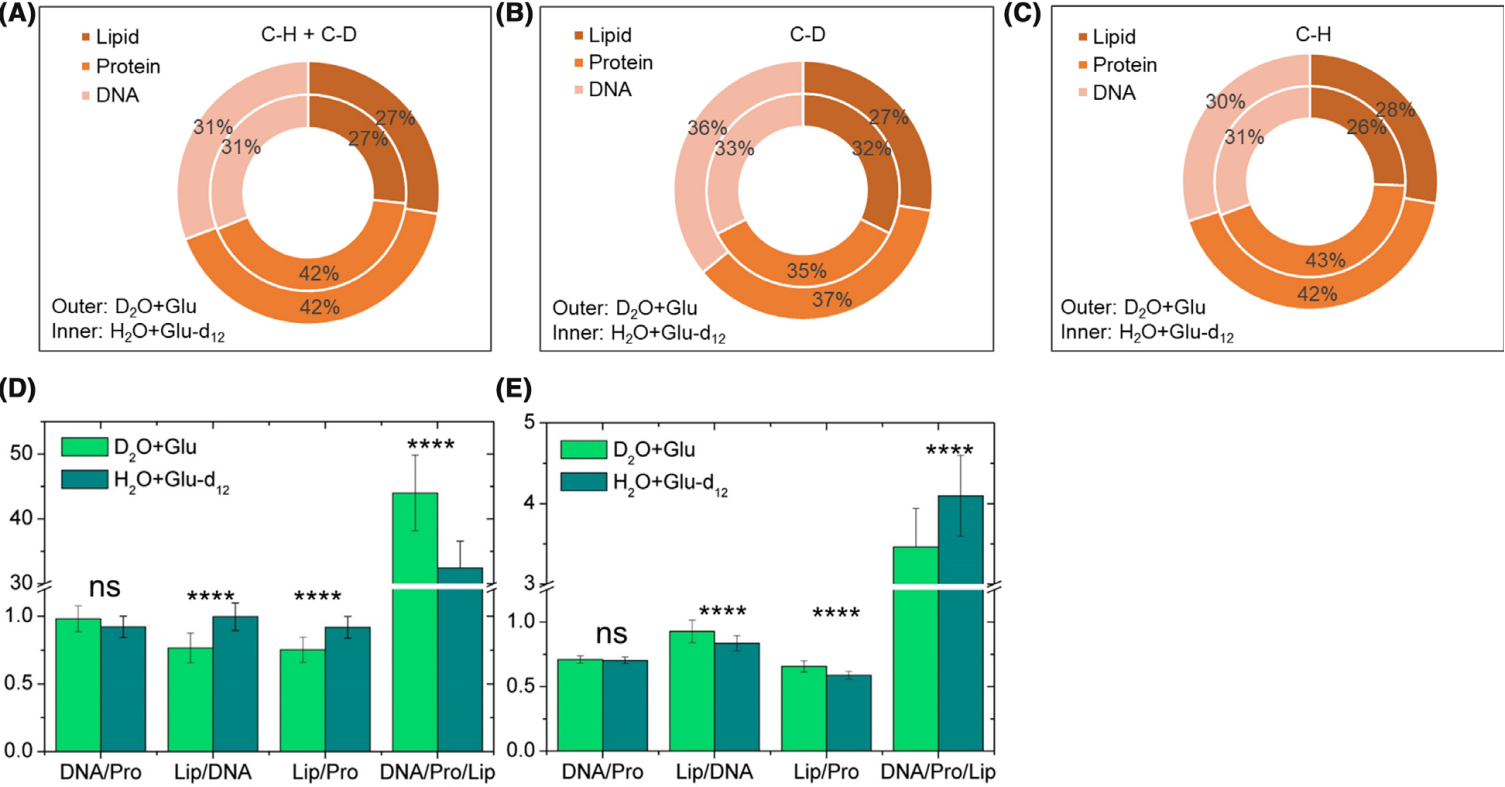
Relative intensities of lipid, protein and DNA were quantified by calculating (A) the sum of the C–D and the C–H band, (B) the C–D band (2109, 2160 and 2220 cm^−1^) and (C) the C–H band (2874, 2928 and 2962 cm^−1^). The ratios of the three components in (D) the C–D band and (E) the C–H band. Student *t*‐test was used to indicate statistical differences between glucose and glucose‐d_12_ conditions, in which ns indicates non‐significant, *P < 0.05, **P < 0.01, ***P < 0.001, ****P < 0.0001.

This spectral observation was further confirmed by quantifying relative intensities of the three components in the C–D band (2109, 2160 and 2220 cm^−1^), C–H band (2874, 2928 and 2962 cm^−1^) and sum of C–D and C–H components (Fig. 4A–C). The sum of C–D and C–H integrals shows similar percentages of lipids, proteins and DNA in D_2_O + glucose and H_2_O + glucose‐d_12_ conditions, indicating an overall consistent cellular content (Fig. 4A). The proportion of lipids increases from 27% to 32% at the C–D region using glucose‐d_12_ compared that using D_2_O (Fig. 4B). This is complementary to a decrease from 28% to 26% of the lipid C–H (Fig. 4C). The DNA and protein C–D with glucose‐d_12_ decrease from 36% to 33% and 37% to 35% respectively, with corresponding increases in the C–H region (Fig. 4B,C).

We then calculated the ratio of DNA/proteins, lipids/DNA, lipids/proteins and DNA/proteins/lipids in the C–D (Fig. 4D) and C–H region (Fig. 4E). The ratio of DNA to proteins, indicative of bacterial replication rate (Xu *et al.*, 2019), shows no significant differences between D_2_O + glucose and H_2_O + glucose‐d_12_ conditions, suggesting similar physiological states of bacteria with the presence of either D_2_O + glucose or H_2_O + glucose‐d_12_. On the other hand, the ratios of lipids/DNA, lipids/proteins and DNA/proteins/lipids all exhibited significant changes at both C–D and C–H regions (Fig. 4D,E). This indicates that the DNA and protein did not exhibit significant change when the lipids were not taken into account. However, when the lipids were considered, the ratio showed a significant difference. Therefore, our results unveil that the D atoms in glucose‐d_12_ have a preference of incorporating into lipids because glucose was metabolized to acetyl‐CoA which can be directly converted into lipids in the human microbiota.

**Figure 5:**
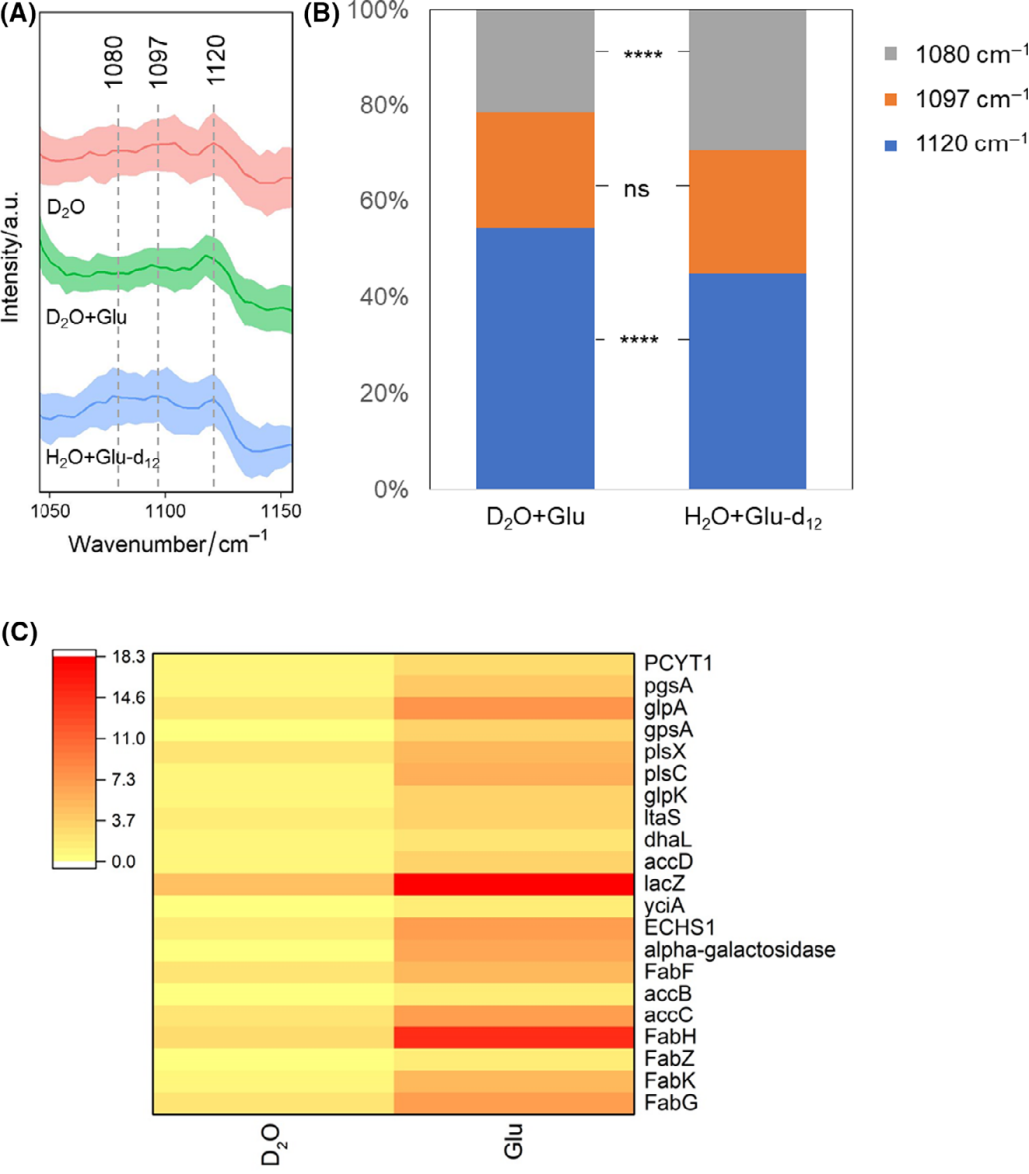
A. Enlarged view of the Raman band of saturated fatty acid shifted from 1120 to 1080 cm^−1^ in the fingerprint region of microbiota cultured *in situ* with D_2_O, D_2_O + 10 mM glucose or H_2_O + 10 mM glucose‐d_12_. B. Quantification of 1120, 1097 and 1180 cm^−1^ band s. Student t‐test was used to indicate statistical differences between glucose and glucose‐d_12_, in which ns indicates non‐significant, *P < 0.05, **P < 0.01, ***P < 0.001, ****P < 0.0001. C. Abundance of gene types associated with lipid synthesis pathways.

In addition to the lipid shift in the C–D region, we also observed a shift in the fingerprint region at 1050–1150 cm^−1^ when cells were cultured with glucose‐d_12_ (Fig. 5A). Raman band at 1097 and 1120 cm^−1^ is assigned to C‐O/C‐C stretching of the carbohydrates (Wiercigroch *et al.*, 2017) and C‐C skeletal stretching of saturated fatty acid (Czamara *et al.*, 2015). A visible increase in the Raman band intensity at 1080 cm^−1^ was observed. Quantitation of the three bands shows there were no changes at 1097 cm^−1^ (carbohydrates), a significant decrease at 1120 cm^−1^ (saturated fatty acid) and a significant increase at 1080 cm^−1^ (Fig. 5B). This result suggests that the band at 1080 cm^−1^ is shifted from the saturated fatty acids band at 1120 cm^−1^ when cells were incubated with glucose‐d_12_, in agreement with the observation of increased lipid content in the C–D region. Moreover, Raman spectra of deuterated dipalmitoyl phosphatidylcholine (DPPC) show similar shifts at 1000–1150 cm^−1^ region in a previous study (Bunow and Levin, 1977). DPPC contains two palmitic acids, which is a kind of saturated fatty acid to store energy and as a precursor to synthesize longer fatty acid chains (Stiebing *et al.*, 2014). It is in an agreement with previous studies that intestinal microbes play significant roles in fermenting monosaccharides to fatty acid (Polan *et al.*, 1964, Tremaroli and Backhed, 2012, den Besten *et al.*, 2013, Kishino *et al.*, 2013).

Figure 5C shows the abundance of 21 genes associated with lipid synthesis in faecal samples with D_2_O and with deuterated glucose according to KEGG database. The averaged abundance of these gene types with glucose addition including *FabF, FabG, FabH, FabK, FabZ, accB, accC and accD* is more than twice as high as that with an absence of glucose. Among the eight gene types, *FabF*, *FabG*, *FabK* and *FabZ* in fatty acid metabolic pathway are responsible for producing hexadecanoyl‐[acp] and octadecanoyl‐CoA in cytoplasm and the key to synthesize common saturated fatty acids such as palmitic acid. The abundance of *ECHS1* is increased by 4.6 times by adding glucose. *ECHS1* participates in producing hexadecanoyl‐CoA in mitochondria during fatty acid metabolism. Both hexadecanoyl‐CoA in mitochondria and hexadecanoyl‐[acp] in cytoplasm are able to produce palmitic acid. Additionally, *YciA* is the key gene to produce palmitic acid in the pathway of biosynthesis of fatty acids. The analysis of functional genes confirmed the results of Raman–DIP that the intestinal bacteria preferentially converted glucose into saturated fatty acids, such as palmitic acid. Additionally, the increased abundance of lipid synthesis genes (Fig. 5C) also may be caused by the increased abundance of the species (Fig. 2C) such as *E. coli*, because metagenomic sequencing measures the genes of all the bacteria in a whole community. On the other hand, Raman–DIP has the ability to detect the phenotype of bacteria at the single‐cell level. Consequently, metagenomic sequencing associated with Raman–DIP is a powerful approach to study microbiota, especially in complex compartments.

### Raman–DIP suggested a different pathway to synthesize aromatic amino acids in gut microbiota

**Figure 6:**
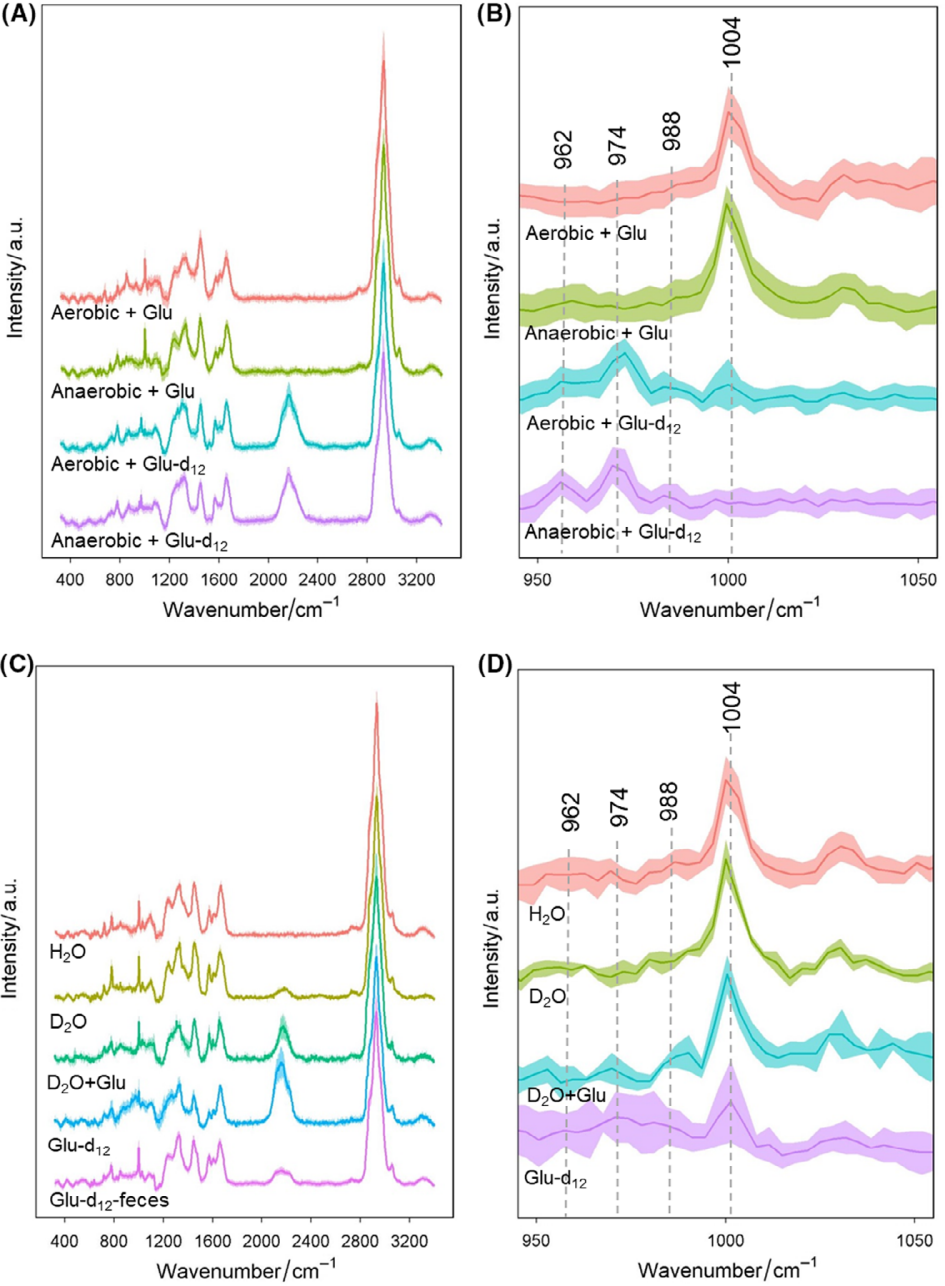
A. SCRS of *Escherichia coli* MG1655 cultured with 10 mM glucose or 10 mM glucose‐d_12_ in aerobic or anaerobic condition. B. Enlarged view of phenylalanine shifts from 1004 to 988, 976 and 962 cm^−1^ at the fingerprint region. C. SCRS of strains isolated from the faeces and cultured with H_2_O, D_2_O, D_2_O + glucose or glucose‐d_12_ in anaerobic condition, compared with the SCRS of bacteria in faecal environment with 10 mM glucose‐d_12_. D. Enlarged view of phenylalanine shifts of the isolated bacterium. Each spectrum represents an average of SCRS from 30 single cells, and the shadow represents standard deviation of SCRS.

The pathway of glucose metabolism into lipids in human intestinal is different from previous studies (Selvarasu *et al.*, 2010; Xu *et al.*, 2017) where catabolism of glucose ended up with a synthesis of aromatic amino acid phenylalanine in cultured *E. coli* DH5α. To determine the effect of the absence of oxygen in the culturing condition, we measured the SCRS of *E. coli* MG1655 cultured aerobically and anaerobically with deuterated glucose, showing in Fig. 6A. In addition to the C–D shifts, red shifts were observed at the phenylalanine band at 1004 cm^−1^ in the fingerprint region (Fig. 6B). Phenylalanine Raman band shifted from 1004 to 988, 976 and 962 cm^−1^ when glucose‐d_12_ were used as the sole carbon source in both aerobic and anaerobic conditions. The result shows that glucose can be transformed into phenylalanine by *E. coli* MG1655 through the shikimate pathway in both aerobic and anaerobic condition. This suggests that the pathway of glucose to synthesis phenylalanine is independent of the absence of oxygen.

We then isolated *Enterococcus *sp., *Clostridium *sp. and *Lactobacillus *sp. from the faeces, which was cultured with H_2_O, D_2_O, D_2_O + glucose and H_2_O + glucose‐d_12_ in anaerobic condition. SCRS of the isolated bacterium with different conditions illustrate various spectral features and C–D intensities (Fig. 6C). Interesting, isotopic shifts of phenylalanine from 1004 to 988, 976 and 962 cm^−1^ were also observed in the isolated strains (Fig. 6D) when culturing with glucose‐d_12_, consistent with the result of *E. coli* MG1655. This suggests that the lipid synthesis pathway from glucose is environment‐dependent in *Enterococcus *sp., *Clostridium *sp. and *Lactobacillus *sp. The intestinal bacteria would convert glucose to lipids only when they are in the microbiota community and loss/reduce the ability when the isolates grow in the laboratory environment as pure species (Fig. S3). Our results suggest that Raman–DIP is able to *in situ* detect the metabolic pathway of microbiota, especially in a complex environment, by analysing isotopic shifts of special Raman bands in SCRS.

By using single‐cell Raman–DIP, we found that the intestinal bacteria have different metabolic rates in the same ecological environment, and some silent members of bacteria can be stimulated by the addition of glucose. Additionally, the catabolic pathway of glucose in human intestine preferentially synthesizes fatty acids such as palmitic acid, which is a different pathway to synthesis aromatic amino acids in pure cultures, assumedly due to the specific environment of gut microbiota and is subject to host specificity.

This study confirms that Raman–DIP is capable to identify the metabolic activity by labelling bacterial cells with D_2_O and recognize the metabolic pathway by culturing the samples in deuterated substrates at the single‐cell level, despite the complexity of compartments such as microbial cells living with soil, plants, animals and humans. The C–D band is shown to be a sensitive Raman marker to indicate the general metabolic activity as well as the substrate utilization by degradation it into different biocomponents. This method can be universal and versatile by harnessing various deuterated substrates to investigate metabolic pathways of tyrosine compounds in a complex environment at the single‐cell level and to explore the subcommunity stimulated by the substrate.

## Experimental procedures

### Sample processing, bacterial strain and growth conditions

Fresh faeces from two healthy human volunteers (no antibiotic treatment 1 year before inclusion) were collected into a culturing tube in anaerobic chamber and 1 g of the sample was dissolved in 10 ml phosphate buffer saline (PBS) solution with 40% of D_2_O (v/v) (Sigma‐Aldrich, St. Louis, MO, USA) or dH_2_O (no‐amendment control). Exogenous nutrient of 10 mM d‐glucose, d‐glucose‐d_12_, tyrosine, tryptophan or oleic acid (Sigma‐Aldrich) was added as a substrate. The samples were homogenized by vortexing and cultured *in situ* at 37°C anaerobically (95% N_2_: 5% H_2_) with shaking (140 rpm). One ml sample was collected at different time points of cultivation (0, 8, 24 and 48 h).

Faecal samples were diluted 1000–10 000 times depending on the cell concentrations and 50 μl of the samples was spread onto LB agar plates. After incubating at 37°C anaerobically for 48 h, several colonies in LB plate were isolated randomly and sent for sequencing (Novogene, Nanjing, China). *Enterococcus *sp., *Clostridium *sp. and *Lactobacillus *sp. were used in this study.

Pure cultures of *E. coli* MG1655 were used as a model species to test the glucose metabolic pathway in M9 minimal medium with 40% D_2_O (v/v) and 10 mM d‐glucose‐d_12_ as the sole carbon source at 37°C anaerobically or aerobically with shaking (140 rpm).

Prior to SCRS measurement, all samples were vortexed for 20 s to homogenize and centrifuged once (300 *g*, 1 min) to remove sediment debris. The supernatant was then centrifuged (8000 *g*, 3 min) to obtain microbial pellets. The cells were resuspended with deionized water three times to remove the impurities and culture medium. For each sample, three replicates were taken.

### Single‐cell Raman spectra measurements and analysis

Samples were diluted to observe single cells under a 100×/0.9 microscope. In each sample, 1.5 μl suspension was spread onto an aluminum (Al)‐coated slide for Raman measurement, as Al gives minimal Raman background. SCRS were obtained using a 532‐nm neodymium‐YAG (yttrium aluminum garnet) laser with a 300 grooves mm^−1^ diffraction grating. SCRS were acquired in the range of 400–3200 cm^−1^ with 5 mW power and 10 s acquisition time. Each faecal sample was measured with 50–80 single‐cell replicates, and 30 single cells were measured for pure cultures.

All spectra were recorded and preprocessed with labspec 6 software (Horiba, Northampton, UK) by baseline correction and vector normalization using the whole spectral range. The quantification of individual Raman bands in SCRS was carried out by integrating the Raman bands at specific wavenumbers by labspec 6 (Horiba). The analysed Raman bands were lipids (2109 cm^−1^), proteins (2160 cm^−1^) and DNA (2220 cm^−1^) at the C–D region, lipids (2874 cm^−1^), proteins (2928 cm^−1^) and DNA (2962 cm^−1^) at the C–H region and deuterium shifted bands at saturated lipids (1080 cm^−1^), carbohydrates (1097 cm^−1^), saturated lipids (1120 cm^−1^) and phenylalanine (962, 976, 988 and 1004 cm^−1^). The integral values can be exported to Excel. The ratio of C–D/(C–D + C–H) was used to indicate the extent of D incorporation from D_2_O or glucose‐d_12_. Statistics and figures were done by using in‐house r scripts (Boston, MA, USA), originlab (Northampton, MA, USA), chemdraw (Akron, OH, USA) and Excel.

### DNA extraction and sequencing

Samples were kept at −80°C until DNA extraction with the MP FastDNA Spin Kit (Loughborough Leicester, UK) for Soil (116560‐200), following the manufacturer's instructions. The extracted DNA was sent to sequencing (Novogene, China) on the HiSeq X platform to generate paired‐end 150 bp reads with 8 bp index sequence.

Raw reads were processed by removing the low‐quality reads with an average quality score below 20, or with 15‐bp adapter sequences, or with 10% or more ambiguous nucleotides. The clean reads were assembled by megahit (v1.0.3; HKU‐BGI Bioinformatics Algorithms Research Laboratory & Department of Computer Science, Hong Kong) with default parameters, and contigs shorter than 200 bp were discarded (Li *et al.*, 2016). The filtered contigs were grouped into different genomes using maxbin (Wu *et al.*, 2016), high‐quality genomes were predicted by metagenemark (v 3.38) (Zhu *et al.*, 2010) and cd‐hit‐est (v4.5.8) (Fu *et al.*, 2012) with a minimum similarity of 95% was applied to de‐redundancy. Then, the clean reads were aligned to the gene set using blast (Krauthammer *et al.*, 2000), the numbers of matched reads were counted and the abundance of each gene was calculated (Qin *et al.*, 2012). The functional gene annotation was used diamond to align KEGG and NCBI nr database with *e* value 10^−5^ (Kanehisa *et al.*, 2006; Buchfink *et al.*, 2015) and the retrieved genomes were annotated using megan (version 5, Tübingen, Germany). The raw data were deposited to NCBI public database with the BioProject number: PRJNA565831.

## Supporting information


**Fig. S1.** (A, B) Structures of the microbial community (top 30 at genus level) of two healthy individuals with different nutrient supplements.
**Fig. S2.** The abundances of stimulated genes catabolizing the compounds of glucose, oleic acid, tryptophan and tyrosine.
**Fig. S3.** Lipids and phenylalanine metabolic pathways from deuterated glucose, in which the yellow arrow indicates the anaerobic pathway, black arrow indicates the aerobic pathway, and red arrow indicates the common pathway.
