# Supplementary material for: Raman–deuterium isotope probing to study metabolic activities of single bacterial cells in human intestinal microbiota
Source: Microb Biotechnol. 2019 Dec 10;13(2):572–83. doi: 10.1111/1751-7915.13519 (PMC7017835; doi:10.1111/1751-7915.13519)
Supplement: Supplementary file 1 — Fig. S1. (A, B) Structures of the microbial community (top 30 at genus level) of two healthy individuals with different nutrient supplements. Fig. S2. The abundances of stimulated genes catabolizing the compounds of glucose, oleic acid, tryptophan and tyrosine. Fig. S3. Lipids and phenylalanine metabolic pathways from deuterated glucose, in which the yellow arrow indicates the anaerobic pathway, black arrow indicates the aerobic pathway, and red arrow indicates the common pathway. [file MBT2-13-572-s001.docx]

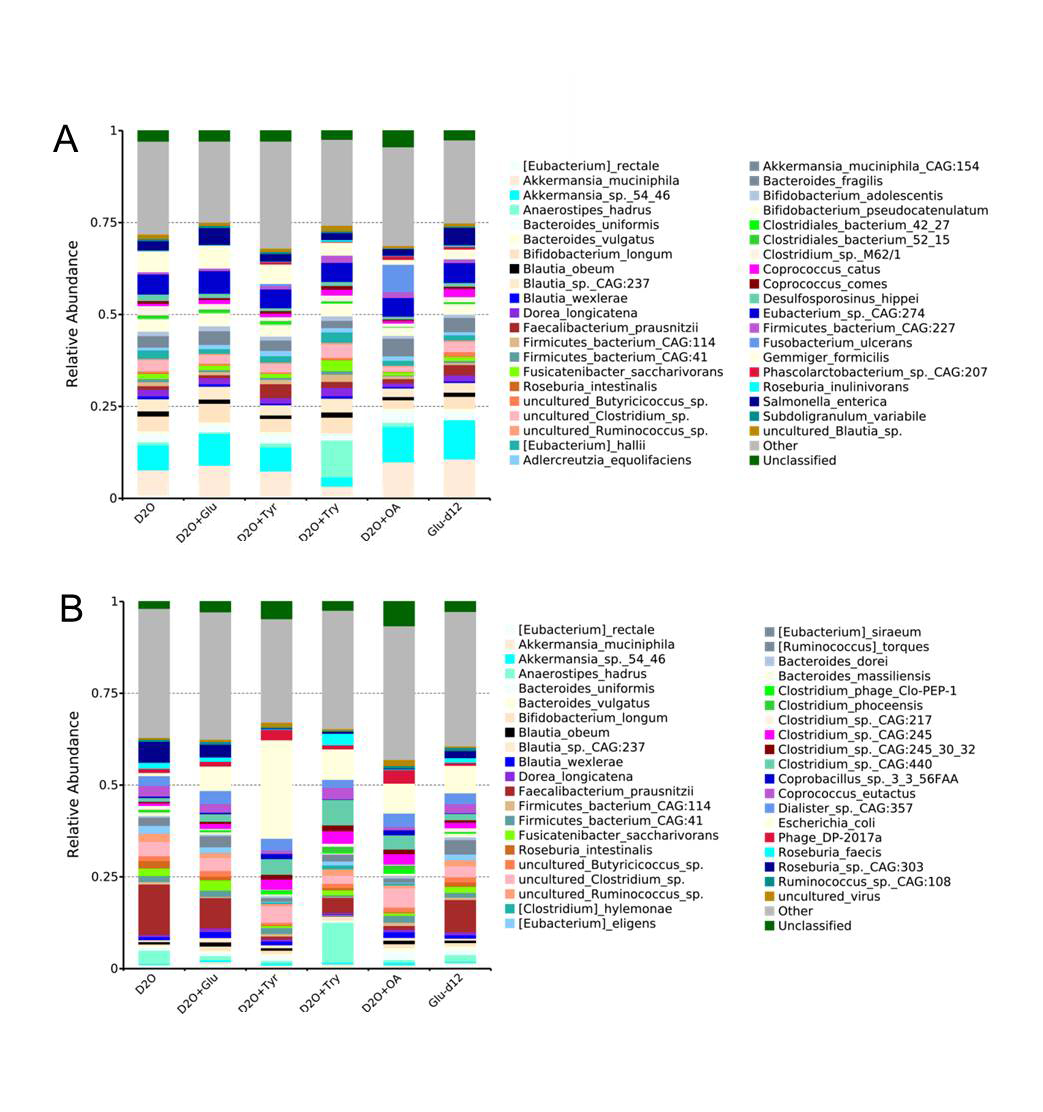


**Figure S1. (A, B) Structures of the microbial community (top 30 at genus level) of two healthy individuals with different nutrient supplements** **(E.coli was excluded).**


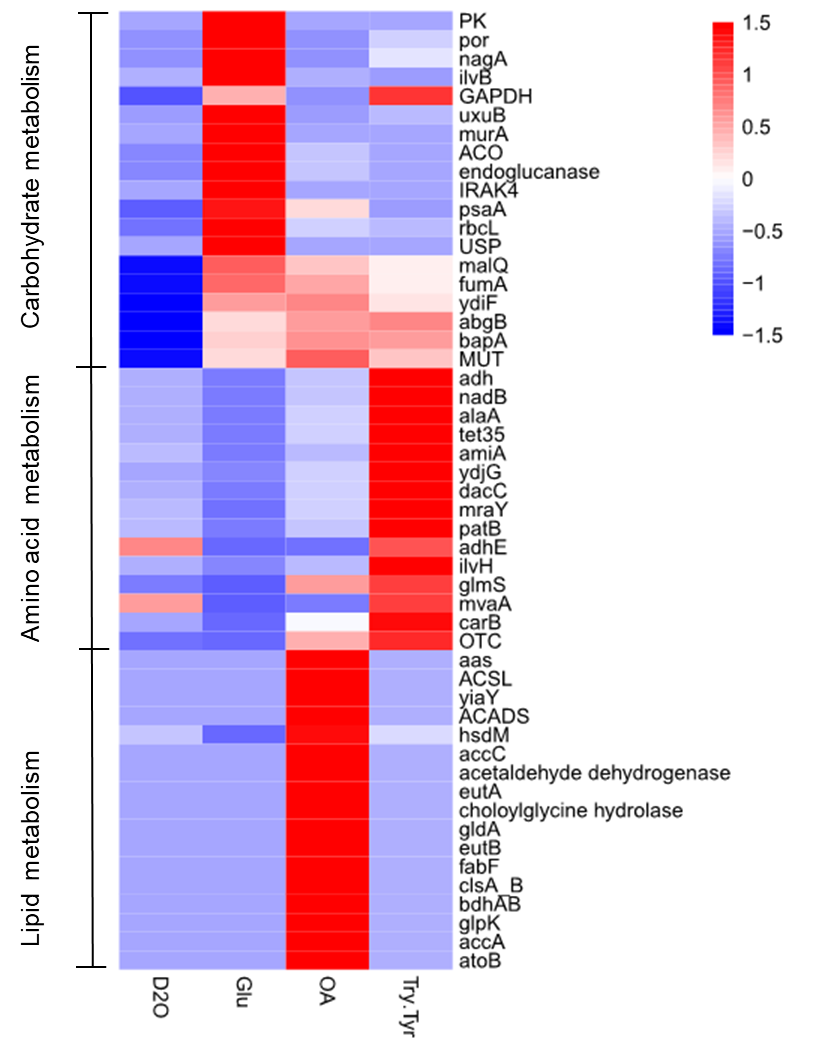


**Figure S2. The abundances of stimulated genes catabolizing the compounds of glucose, oleic acid, tryptophan and tyrosine.**


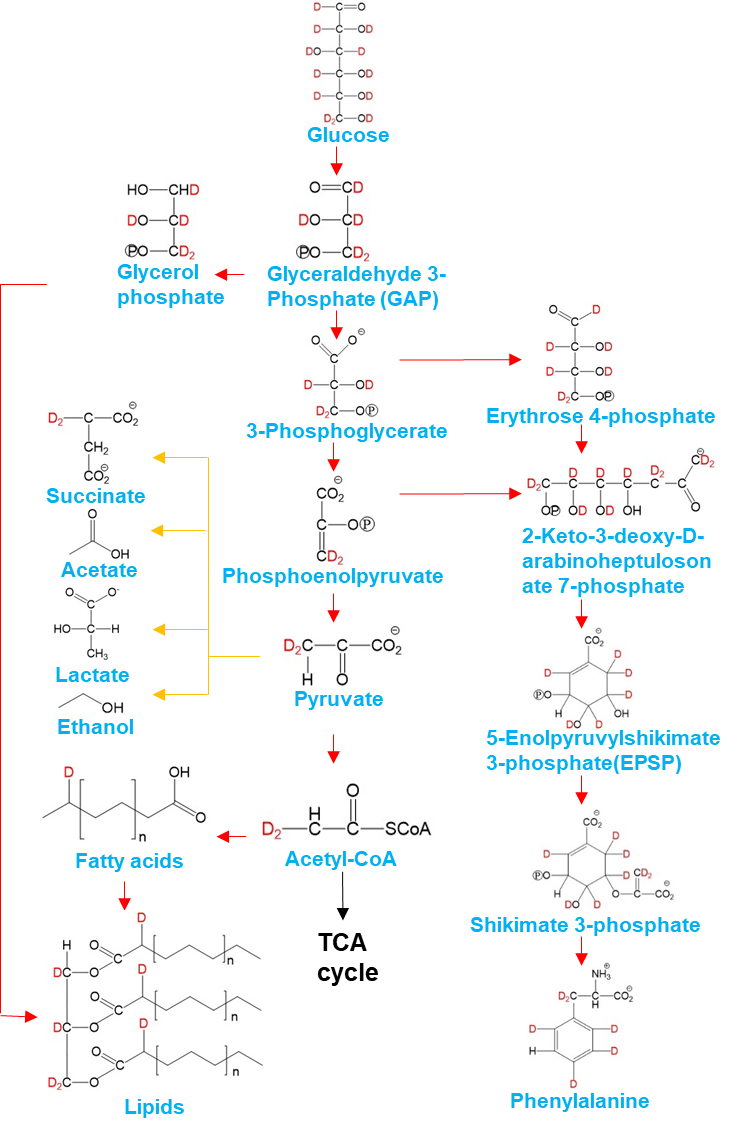


**Figure S3.** Lipids and phenylalanine metabolic pathways from deuterated glucose, in which the yellow arrow indicates the anaerobic pathway, black arrow indicates the aerobic pathway, and red arrow indicates the common pathway. The main metabolic pathway of glucose leads to production of lipids in human guts, while the synthesis of phenylalanine is preferred in cultured *E. coli* MG1655 and DH5α.
